# Gelation Dynamics during Photo-Cross-Linking of Polymer Nanocomposite Hydrogels

**DOI:** 10.1021/acspolymersau.2c00051

**Published:** 2022-12-05

**Authors:** Michael C. Burroughs, Tracy H. Schloemer, Daniel N. Congreve, Danielle J. Mai

**Affiliations:** † Department of Chemical Engineering, Stanford University, Stanford, California94305, United States; ‡ Department of Electrical Engineering, Stanford University, Stanford, California94305, United States

**Keywords:** Nanocomposite hydrogels, photo-cross-linking, rheology, star polymers, nanocapsules, gelation

## Abstract

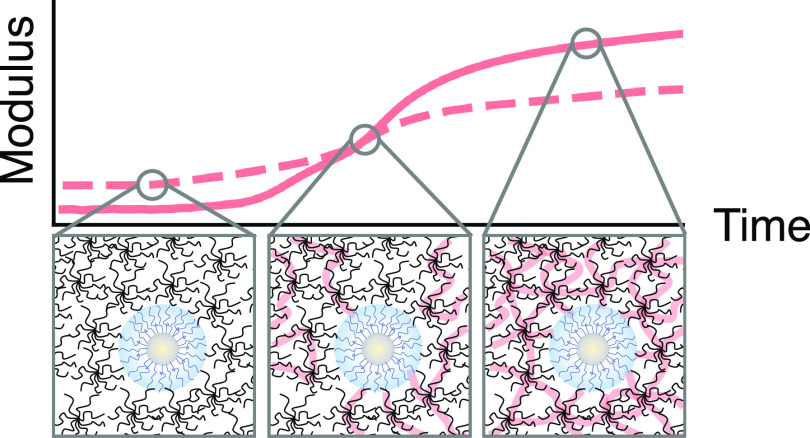

Embedding nanomaterials into polymer hydrogels enables the design of functional materials with tailored chemical, mechanical, and optical properties. Nanocapsules that protect interior cargo and disperse readily through a polymeric matrix have drawn particular interest for their ability to integrate chemically incompatible systems and to further expand the parameter space for polymer nanocomposite hydrogels. The properties of polymer nanocomposite hydrogels depend on the material composition and processing route, which were explored systematically in this work. The gelation kinetics of network-forming polymer solutions with and without silica-coated nanocapsules bearing polyethylene glycol (PEG) surface ligands were investigated using *in situ* dynamic rheology measurements. Network-forming polymers comprised either 4-arm or 8-arm star PEG with terminal anthracene groups, which dimerize upon irradiation with ultraviolet (UV) light. The PEG-anthracene solutions exhibited rapid gel formation upon UV exposure (365 nm); gel formation was observed as a crossover from liquid-like to solid-like behavior during *in situ* small-amplitude oscillatory shear rheology. This crossover time was non-monotonic with polymer concentration. Far below the overlap concentration (*c*/c* ≪ 1), spatially separated PEG-anthracene molecules were subject to forming intramolecular loops over intermolecular cross-links, thereby slowing the gelation process. Near the polymer overlap concentration (*c*/c* ∼ 1), rapid gelation was attributed to the ideal proximity of anthracene end groups from neighboring polymer molecules. Above the overlap concentration (*c*/c* > 1), increased solution viscosities hindered molecular diffusion, thereby reducing the frequency of dimerization reactions. Adding nanocapsules to PEG-anthracene solutions resulted in faster gelation than nanocapsule-free PEG-anthracene solutions with equivalent effective polymer concentrations. The final elastic modulus of nanocomposite hydrogels increased with nanocapsule volume fraction, signifying synergistic mechanical reinforcement by nanocapsules despite not being cross-linked into the polymer network. Overall, these findings quantify the impact of nanocapsule addition on the gelation kinetics and mechanical properties of polymer nanocomposite hydrogels, which are promising materials for applications in optoelectronics, biotechnology, and additive manufacturing.

## 1 Introduction

Advances in photo-cross-linking have broadened the use of polymer hydrogels as tissue engineering scaffolds,^1−4^ antifouling surfaces,^5−7^ and photonic materials.^8−10^ Polymer hydrogels are versatile materials comprising cross-linked polymer networks that are swollen in water. Polymer network formation by photo-cross-linking offers several advantages over conventional approaches such as thermal or redox-initiated cross-linking: it proceeds under mild reaction conditions including ambient temperature and moderate pH, and it enables spatiotemporal control upon tuning light intensity, exposure time, and illumination area.^11,12^ This control is often leveraged to build complex hydrogel structures through additive manufacturing,^13,14^ tunable photodegradation,^15−17^ or surface patterning.^18−20^

Photodimerization reactions further simplify the formulation of photo-cross-linked hydrogels, such that gelation requires only one polymeric precursor. In this approach, hydrophilic polymers are functionalized with photoresponsive groups such as anthracene,^21−24^ cinnamylidene,^25^ coumarin,^26−28^ styrylpyrene,^29,30^ and thymine.^31^ These groups form dimers upon irradiation with high energy light (250–450 nm). This strategy does not require added photoinitiators, reactive monomers, or cross-linking agents beyond the polymeric precursor. Poly(ethylene glycol) (PEG) is a common hydrophilic component for its low-protein-adsorption properties,^32,33^ well-characterized mechanical responses,^34,35^ and commercial availability of multifunctional synthetic precursors.^36^ Anthracene has drawn particular interest as a photoresponsive group that undergoes [4 + 4] cycloaddition upon exposure to low-intensity, near-ultraviolet light (365 nm),^21^ which is suitable for biological systems.^37^ Recently, PEG-anthracene hydrogels were used as matrices with tunable stiffnesses from 0.1–100 kPa; photodimerization enabled on-demand stiffening to mimic cardiac tissue environments.^38^

The mechanical properties of polymer hydrogels can be further tailored by incorporating nanomaterials to form polymer nanocomposite hydrogels.^39,40^ Mechanical reinforcement emerges from the adsorption of polymer chains to nanomaterial surfaces.^41−44^ Nanomaterial–polymer interactions increase the density of effective cross-links throughout the polymer nanocomposite hydrogel, thereby increasing the bulk elastic modulus.^45−48^ Interestingly, nanocomposite stiffness is independent of nanomaterial size and governed by the volume fraction ϕ_nano_ according to the Guth–Gold model:^49^
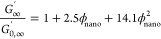
1where *G*_∞_^′^ and *G*_0,∞_^′^ are the elastic moduli of polymer networks with and without added nanomaterials, respectively.

While nanocomposite stiffness is independent of nanomaterial shape and size, judicious nanomaterial selection expands the chemical, electronic, optical, and/or biological functions of polymer nanocomposite hydrogels. For example, nanocapsules enable applications in photonics,^50−52^ drug delivery,^53−55^ and bioimaging^56−58^ by protecting components that are incompatible with their external environments. Recently, Sanders and Schloemer et al. encapsulated triplet-fusion upconversion materials in silica-coated nanocapsules, which were introduced into a photopolymerizable resin to enable volumetric 3D-printing processes.^52^ Unlike conventional extrusion-based approaches to 3D-printing, volumetric 3D-printing is performed by rastering the focal point of incident light through a photocurable resin to initiate localized polymerization.

To guide the broader use of polymer nanocomposite hydrogels, especially in dynamic applications such as 3D-printing, it is important to understand the impact of nanocapsules on mechanical properties during and after gelation. *In situ* dynamic rheology captures the evolution of mechanical properties during photo-cross-linking by coupling a shear rheometer to an irradiation fixture. Dynamic rheology measurements of polymer nanocomposites have shown that nanomaterial additives can convolute the conventional analysis of polymer network formation. Nanomaterial–polymer interactions often obscure the rheological signatures of chemical cross-links in the polymeric network,^59−62^ indicating that synergistic nanomaterial–polymer interactions during gelation must be considered for dynamic applications of these materials. The final material properties and microstructure after gelation are also influenced by the overall formulation.^63−65^ Generally, increasing the polymer concentration subsequently increases the network stiffness.^38,62^ In contrast, the impact of nanomaterial concentration is challenging to predict due aggregation or flocculation of nanomaterials,^9,59^ “network dilution” due to defects in the polymer network,^66−68^ or heterogeneous local chemical environments near nanomaterial surfaces.^63^

This work explores the evolution of mechanical properties and gelation dynamics during photo-cross-linking of polymer nanocomposite hydrogels comprising multiarm PEG-anthracene and silica-coated nanocapsules. The hydrodynamic sizes of 4-arm and 8-arm PEG-anthracene in water were measured to estimate the polymer overlap concentration, which guided the formulation of dilute, semidilute, and concentrated polymer solutions with and without silica-coated nanocapsules. *In situ* dynamic rheology upon exposure to 365 nm radiation revealed the influence of the number of arms per polymer, polymer concentration, and nanocapsule concentration on material properties during and after gelation. In the absence of nanocapsules, a non-monotonic dependence of the gel time on PEG-anthracene concentration emphasizes the importance of intermolecular anthracene dimerization on network formation. Nanocapsule incorporation resulted in faster gelation times and increased elastic moduli compared to PEG-anthracene hydrogels. These results suggest the importance of cooperative nanoparticle–polymer interactions to enhance and to customize the final material properties of polymer nanocomposite hydrogels.

## 2 Materials and Methods

### 2.1 Materials

#### 2.1.1 Anthracene Functionalization of Multiarm Star Polymers

9-Anthracenecarboxylic acid (9ACA, CAS 723-62-6, 99%), 1-[bis(dimethylamino)methylene]-1*H*-1,2,3-triazolo[4,5-*b*]pyridinium 3-oxide hexafluorophosphate (HATU, CAS 148893-10-1, 97%), 4-(dimethylamino)pyridine (DMAP, CAS 1122-58-3, >99%), 4-methylmorpholine (CAS 109-02-4, 99%), and anhydrous dimethylformamide (DMF, CAS 68-12-2, 99.8%) were purchased from Millipore Sigma and used as received. Amine-terminated poly(ethylene glycol) (PEG-amine) multiarm star polymers were purchased from Nanosoft Polymers (4 arms, 20 000 g/mol) and JenKem USA (8 arms, 20 000 g/mol).

#### 2.1.2 Nanocapsule Fabrication

Methoxy-poly(ethylene glycol)-silane (mPEG-silane, 10 000 g/mol) was purchased from Nanosoft Polymers. Anhydrous tetraethyl orthosilicate (TEOS, CAS 78-10-4, 98%) was purchased from Acros Organics. Anhydrous (3-aminopropyl)triethoxysilane (APTES, CAS 919-30-2, 99%) was purchased from Millipore Sigma. Oleic acid (CAS 112-80-1, 99%) was purchased from Beantown Chemical. All chemicals were used as received.

### 2.2 Procedures

#### 2.2.1 Polymer Functionalization with Anthracene

**Scheme 1:**
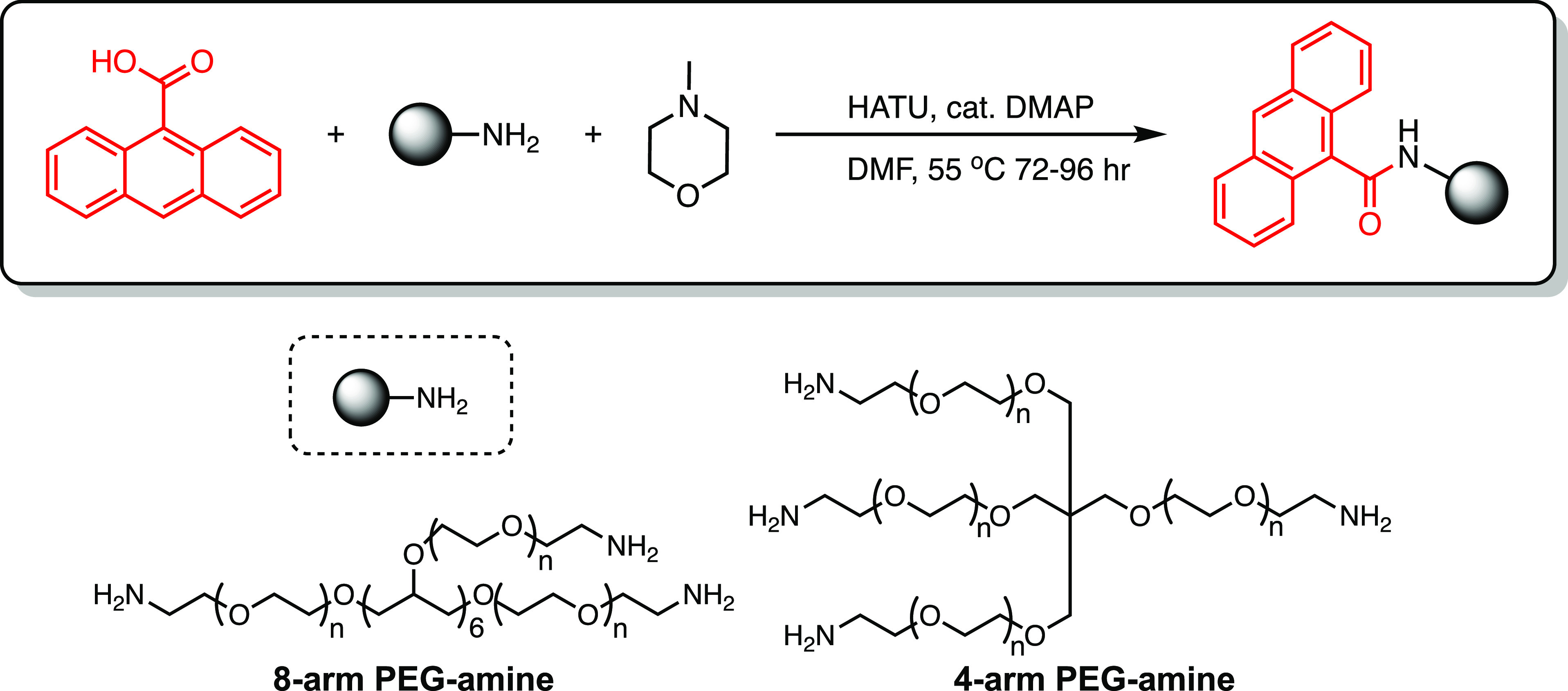
Reaction Scheme for the Formation of Anthracene-Functionalized Multiarm PEG (PEG-Anthracene)

Multiarm anthracene-terminated PEGs (PEG-anthracene) were produced by reacting PEG-amine with 9-anthracenecarboxylic acid (9ACA) (Scheme 1).^23,38^ All equivalents are with respect to the moles of polymer ends. 9ACA (2 equiv), HATU (4 equiv), PEG-amine (0.500 g), and DMAP (cat.) were added to a 25 mL Schlenk flask with a stir bar. The flask was evacuated and backfilled with nitrogen 3× prior to the addition of anhydrous DMF (10 mL) and 4-methylmorpholine (4 equiv). The reaction stirred for 72–96 h at 55 °C under nitrogen in the dark (under aluminum foil). The reaction was allowed to cool to room temperature and transferred to dialysis tubing (3500 MWCO, regenerated cellulose, Fisher Brand). Samples were dialyzed against DMF in a fume hood (800 mL, 4 cycles, at least 6 h/cycle) followed by ultrapure water (Milli-Q, 18.2 MΩ-cm, 800 mL, 3 cycles, at least 6 h/cycle). Finally, the aqueous polymer solution was frozen at −80 °C and subsequently lyophilized to yield a light-yellow powder. The final yields of 4-arm and 8-arm PEG-anthracene were 0.441 g (85%) and 0.514 g (95%), respectively, as confirmed by ^1^H NMR (Supporting Information, Figures S1 and S2). As a safety precaution, butyl gloves were worn outside of nitrile gloves when handling large volumes of DMF during dialysis.

#### 2.2.2 Nanocapsule Fabrication

Silica-coated nanocapsules containing oleic acid were fabricated according to established procedures.^52^ Briefly, 200 mL of prechilled ultrapure water (∼5 °C) was added to a Vitamix blender (Vitamix E310 Explorian Blender, Professional grade, 48 oz) in a nitrogen-filled glovebox. Then 1.45 mL of oleic acid was added to the ultrapure water, and the solution was blended at the maximum speed for 60 s. The oleic acid/water emulsion was transferred to a 500 mL doubled-necked, round-bottom flask, under constant stirring at 1200 rpm. APTES was added until the mixture became transparent (typically 0.75 mL). mPEG-silane (4 g) was added immediately upon this transition from opaque to transparent to prevent capsule aggregation. Anhydrous TEOS (30 mL) was then added at once to facilitate growth of the silica shell. The flask was then capped under continuous stirring and removed from the glovebox after 10 min. The flask was then connected to a Schlenk line and stirred at 1200 rpm at T = 65 °C under constant nitrogen pressure. After 42 h, a second addition of mPEG-silane (4 g) was added to the reaction mixture. After 48 h, the reaction mixture was allowed to cool to room temperature and transferred to a centrifuge tube. The mixture was centrifuged at 8670*g* (Lynx Sorvall Ultracentrifuge) for 1 h to remove larger aggregates. The resulting pellet was discarded, and the supernatant was centrifuged for an additional 12–14 h at 8670*g* to concentrate the silica-coated nanocapsules. The second centrifugation step produced approximately 8–10 g of a nanocapsule-rich paste-like substance, which was immediately dispersed in ultrapure water.

#### 2.2.3 Sample Preparation

##### 2.2.3.1 Polymer Solutions

PEG-amine and PEG-anthracene solutions were prepared by dissolving desired masses of dry polymer in fixed volumes of ultrapure water. Reported concentrations (mg/mL) account for the polymer volume by assuming a polymer density of 1.125 g/cm^3^. For example, a 100 mg/mL solution was prepared by adding 90 mg of dry polymer to 0.82 mL of water. Solutions were vortex mixed for at least 1 min to ensure full dissolution of polymers.

##### 2.2.3.2 Polymer Nanocomposite Solutions

Polymer nanocomposite solutions were prepared by dissolving desired masses of dry polymer in fixed volumes of a dilute nanocapsule dispersion and ultrapure water. The mass of nanocapsules in the dispersion was determined by thermal gravimetric analysis (TGA, TA Instruments TGA Q5500) prior to preparing polymer nanocomposite solutions. The densities of PEG-anthracene (1.125 g/cm^3^) and silica-coated nanocapsules (1.44 g/cm^3^) were used to formulate polymer nanocomposite solutions with known volume fractions.

#### 2.2.4 Dynamic Light Scattering

Dynamic light scattering (DLS) was used to quantify the hydrodynamic radii of star polymers and nanocapsules. DLS was performed using a Brookhaven NanoBrook Omni with a 640 nm diode laser and a fixed temperature of 22 °C. Scattered photon counts were detected at 90° relative to the incident beam path. Prior to measurement, aqueous PEG-amine, PEG-anthracene, and nanocapsule solutions were diluted to 10 mg/mL, 10 mg/mL, and 40 mg/mL, respectively. Solutions were passed through 0.2 μm poly(ether sulfone) (PES) syringe filters directly into prerinsed and nitrogen-dried 1.5 mL polystyrene cuvettes. The hydrodynamic radii of star polymers and nanocapsules in water were quantified by averaging across 3 consecutive DLS measurements (5 min scans).

#### 2.2.5 Rheological Characterization

*In situ* cross-linking dynamics were monitored using small-amplitude oscillatory shear measurements during sample exposure to ultraviolet (UV) light on a stress-controlled TA Instruments Discovery HR 30 rheometer. The rheometer was fitted with an optics plate accessory (OPA; TA Instruments) bearing a custom-ordered quartz disc (76.2 mm diameter and 1.57 mm thick, Technical Glass Products, Inc.). The OPA facilitated mounting of a collimated 365 nm UV LED lamp (435 mW, M365LP1-C2; ThorLabs) below the quartz disc to enable sample irradiation during rheological measurements; see rheo-optical setup in Figure S3. The light intensity was controlled with an analog LED driver (LEDD1B; ThorLabs). UV intensities at the sample location were set to 2.7 mW/cm^2^ for photo-cross-linking experiments as measured using a power meter; a UV intensity calibration curve is shown in Figure S4. Temperature control was provided by an Upper Peltier Plate accessory (UPP; TA Instruments) with 25 mm diameter disposable aluminum parallel plates (TA Instruments). A tight-fitting light jacket fully enclosed the sample and eliminated evaporative sample losses.

*In situ* rheological measurements were performed to quantify the kinetics of network formation during anthracene dimerization. The sample thickness was fixed at 100 μm for all experiments to ensure uniform irradiation of samples and to reduce the required penetration depth of UV light; an estimation of UV light attenuation is included in the Supporting Information.^69^ All experiments were performed at a fixed temperature of 22 °C. Samples were presheared at 10 rad/s for 60 s to eliminate loading hysteresis followed by a 60 s equilibration period. Oscillation time sweeps were performed at 10 rad/s with a strain amplitude of 10%. The oscillation frequency corresponds to a data sampling frequency of approximately 1 datum every 6 s. The strain amplitude provided sufficient torque signals to measure low-viscosity polymer solutions at early times and to probe final hydrogels in the linear viscoelastic regime (Figure S5). To generate a baseline for polymer solutions prior to cross-linking, samples were not exposed to UV irradiation until 60 s after the onset of the oscillation time sweep. Frequency sweeps spanning 0.1 to 100 rad/s were also performed before and after UV irradiation of PEG-anthracene solutions and nanocomposites (Figures S6–S8).

## 3 Results and Discussion

### 3.1 Determination of the Overlap Concentration

To guide the rational formulation of polymer nanocomposite hydrogels, the overlap concentration c* was determined for multiarm PEG-anthracene in water. c* represents the concentration at which swollen, space-filling polymer molecules begin to interact with each other in solution. Below c*, the polymer solution is dilute, and individual polymer molecules are considered noninteracting. Above c*, the polymer solution is sufficiently concentrated, such that neighboring polymer molecules undergo intermolecular interactions that dominate the physical properties of the solution. The exact value of c* depends on polymer size, specifically the weight-average molecular weight *M*_w_ and radius of gyration *R*_g_:
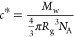
2where *N*_A_ is Avogadro’s number.^70,71^

**Figure 1:**
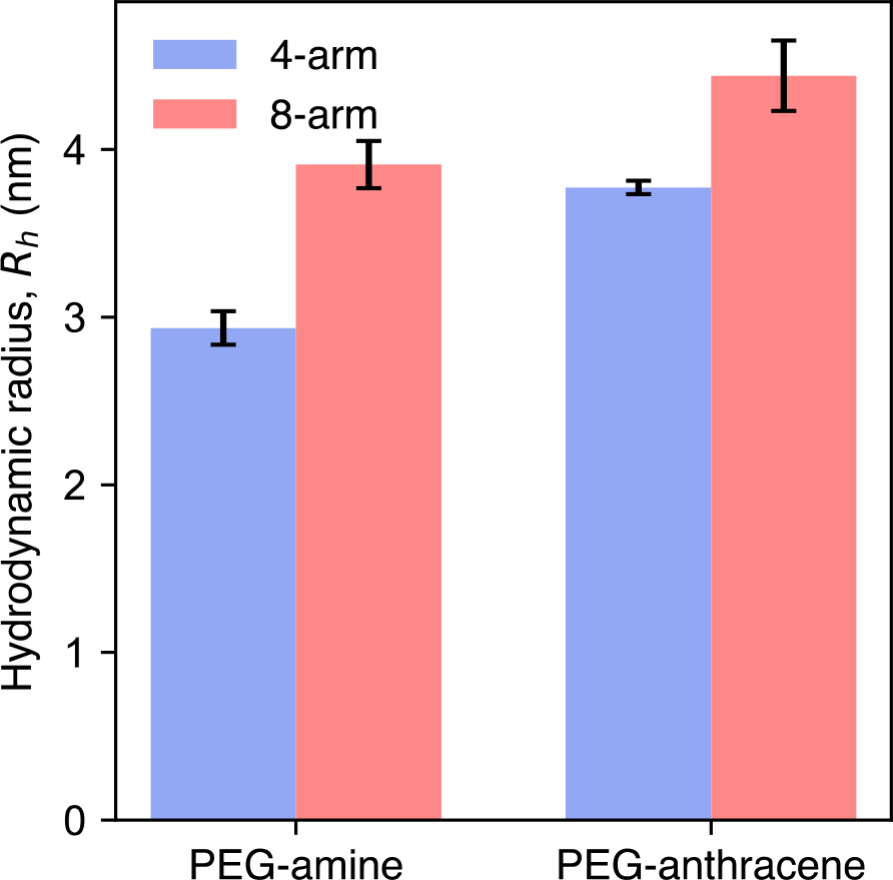
Anthracene functionalization increases the hydrodynamic radii *R*_h_ of multiarm PEG in water at 22 °C. For unfunctionalized (PEG-amine) and functionalized (PEG-anthracene) star polymers of equal molecular weight (20 000 g/mol), *R*_h_ is larger for 8-arm polymers compared to 4-arm polymers. Error bars indicate standard error (*n* = 3).

For multiarm PEG-amine and PEG-anthracene with equal molecular weights (20 000 g/mol) and different numbers of arms, hydrodynamic radii *R*_h_ were measured by dynamic light scattering as proxies for *R*_g_. Although *R*_g_ and *R*_h_ are distinct physical parameters, these quantities are nearly equivalent for 4- and 8-arm star polymers (within 5%).^70^Figure 1 shows that 8-arm polymers are larger than 4-arm polymers for both PEG-amine and PEG-anthracene. The arm-number dependence of *R*_h_ is consistent with the scaling relationship for multiarm star polymers:^72^
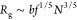
3where *b* is the Kuhn length, *f* is the number of arms, and *N* is the total number of Kuhn monomers. According to this scaling relationship, 8-arm star polymers should be approximately 15% larger than 4-arm star polymers of equal molecular weight, which is consistent with the 17% increase in *R*_h_ for 8-arm PEG-anthracene (4.4 ± 0.2 nm) compared to 4-arm PEG-anthracene (3.77 ± 0.04 nm). The hydrodynamic radii of 4- and 8-arm PEG-anthracene corresponded to overlap concentrations of 140 and 90 mg/mL, respectively. *R*_h_ also increased upon functionalization of multiarm PEG-amine to PEG-anthracene. The larger radius of PEG-anthracene relative to PEG-amine is attributed to the hydrophobic nature of anthracene end groups, such that hydrophilic PEG arms swell to improve overall solubility in water.

### 3.2 Gelation Dynamics of PEG-Anthracene

**Figure 2:**
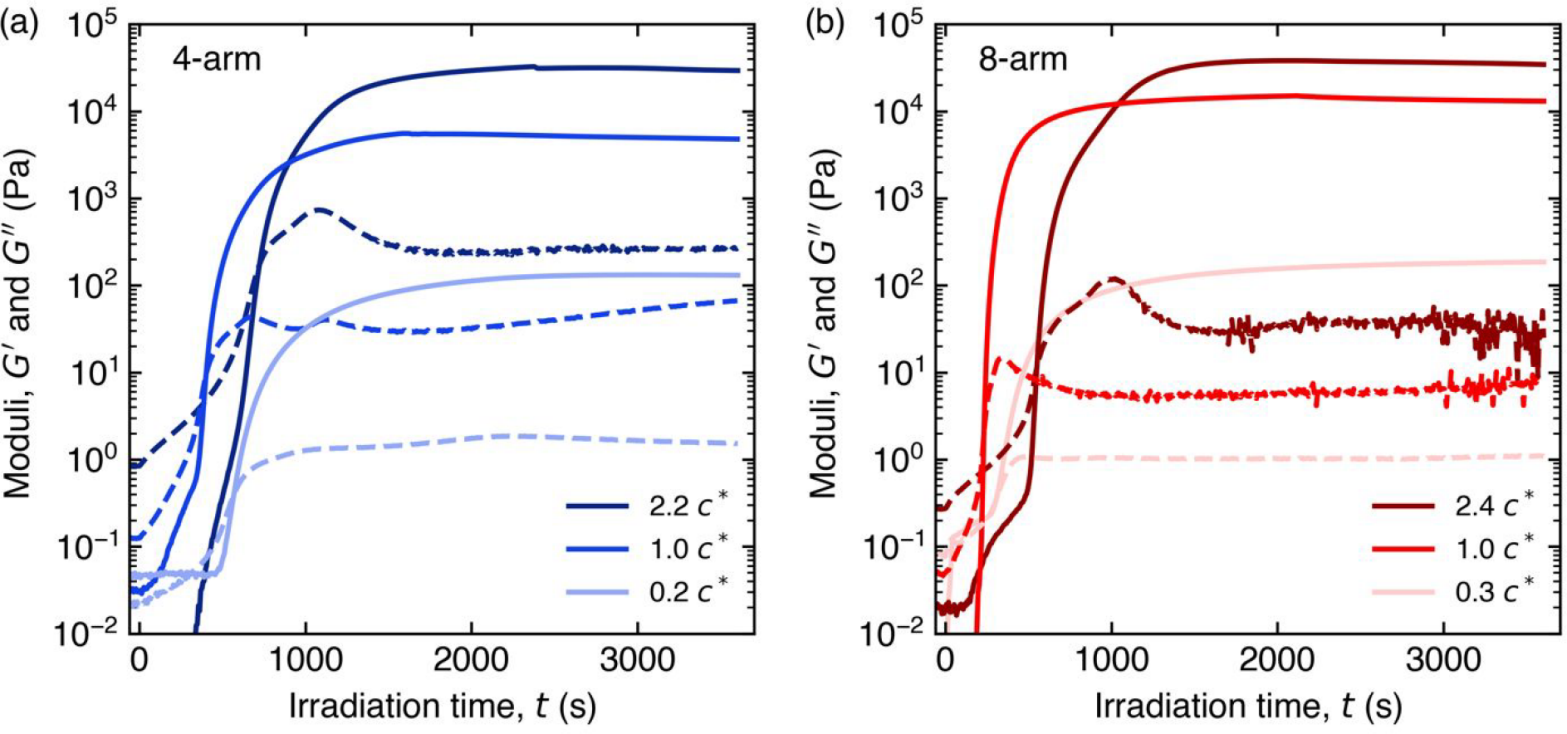
*In situ* small-amplitude oscillatory shear measurements reveal cross-linking of (a) 4-arm and (b) 8-arm PEG-anthracene hydrogels upon irradiation with 365 nm UV light (2.7 mW/cm^2^) at 22 °C. Solid lines (—) denote the elastic modulus, dashed lines (---) denote the viscous modulus, and each shade indicates a different concentration of PEG-anthracene. The strain amplitude and oscillation frequency were 10% and 10 rad/s, respectively.

Rheological measurements of PEG-anthracene in water before, during, and after cross-linking provided information about polymer network structure and dynamics. The transient rheological evolution during the gelation of 4- and 8-arm PEG-anthracene solutions was measured using a custom *in situ* rheo-optical instrument, which allows for simultaneous small-amplitude oscillatory shear measurements and UV light irradiation. *In situ* photo-cross-linking experiments demonstrated that the emergence of an elastic response depends strongly on the number of arms and concentration of multiarm star polymers (Figure 2a,b). For all solutions investigated, the elastic (*G*′) and viscous (*G*″) moduli exhibited an initial incubation period upon exposure to UV light, followed by a rapid increase in *G*′ by up to nearly 6 orders of magnitude before reaching a steady-state value. During the period where *G*′ increased rapidly, a crossover between *G*″ and *G*′ indicated a transition from predominantly liquid-like, viscous behavior to predominantly solid-like, elastic behavior. This crossover time or “gel time” (*t*_gel_) is associated with the gel point, at which the polymer network spans the entire sample thickness. Generally, quantifying the time at which the gel point is reached requires use of the Winter–Chambon criterion.^73^ The Winter–Chambon criterion was not employed in this work due to known complications applying the criterion to filled polymer systems.^59^*t*_gel_ was determined by fitting the time-dependent tan(δ) = *G*^″^/*G*′ near the crossover to a single exponential decay function and solving for the time at which tan(δ) = 1 (Figure S9). Following *t*_gel_, *G*′ continued to increase sharply prior to reaching a plateau value at longer times.

The viscous modulus also exhibited dynamic changes during photo-cross-linking. PEG-anthracene solutions initially exhibited viscous-dominant behavior. *G*″ increased upon approaching the crossover between *G*″ and *G*′, and this behavior was attributed to the initial formation and growth of cross-linked clusters of PEG-anthracene during photo-cross-linking. Beyond *t*_gel_, *G*″ reached a maximum before decaying to a steady-state value for concentrations near and above c*. Multiple local maxima were observed in some cases, such as the 4-arm, *c*/c* = 1.0 sample in Figure 2a. Replicate experiments reveal variability in the number and amplitude of these local maxima (Figure S10). The non-monotonic response in *G*″ suggests the incorporation of large, cross-linked polymer clusters into the bulk network. Initially, cluster growth increases the viscosity of the soluble fraction (evident by the increase in *G*″); subsequent incorporation of clusters into the bulk polymer network reduces the viscosity of the soluble fraction. Therefore, the final viscous modulus (*G*_∞_^″^) is attributed to the contributions of unincorporated polymer clusters, elastically ineffective strands (e.g., intramolecular loops and dangling ends), and water. At long times, both *G*″ and *G*′ show no appreciable increase upon continued UV exposure, indicating the formation of a steady-state hydrogel. Despite the smooth surfaces of the disposable upper and quartz lower plates, evidence of slip was not observed during these final stages of photo-cross-linking.

**Figure 3:**
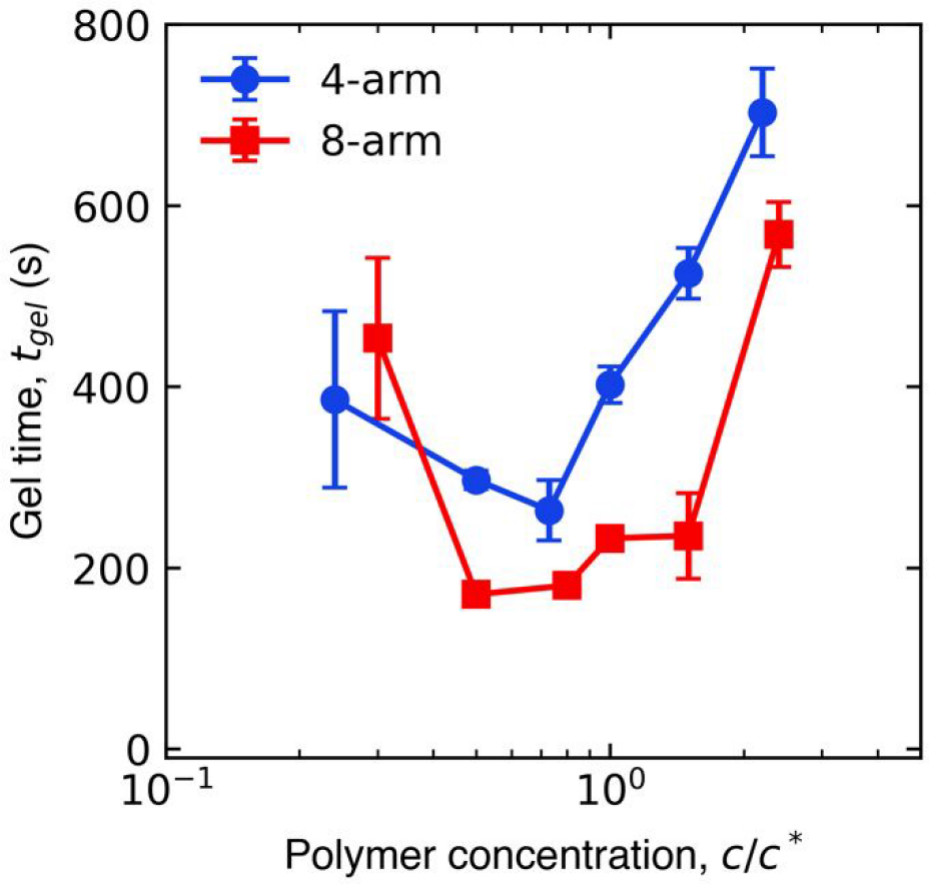
*In situ* dynamic rheology revealed non-monotonic gel times with respect to the concentration of multiarm PEG-anthracene solutions. Gel times were determined as the time of crossover between *G*′ and *G*″ during small-amplitude oscillatory shear measurements. Error bars indicate standard error (*n* = 3), and some error bars are smaller than the symbols.

Gel times were non-monotonic with respect to polymer concentration for both multiarm PEG-anthracene samples (Figure 3). This non-monotonicity reflects different underlying polymer dynamics that depend on solution composition. At concentrations far below c*, dilute star polymers are susceptible to intramolecular anthracene dimerization and the formation of elastically ineffective loops; rare collisions between neighboring star polymers lead to the eventual formation of a weak polymer network. Conversely, at concentrations near c*, intermolecular anthracene dimerization is favored due to the proximity of neighboring star polymer ends. Above c*, increased solution viscosity and intermolecular associations hinder molecular diffusion, thereby reducing the frequency of anthracene dimerization events and resulting in a slower gel time.

Importantly, the minimum gel time near the polymer overlap concentration suggests optimal solution compositions for fast photo-cross-linking of multiarm PEG-anthracene solutions. For most concentrations (*c*/c*) investigated, 8-arm PEG-anthracene solutions reached the gel point faster than 4-arm PEG-anthracene solutions. Faster gel times for 8-arm PEG-anthracene were attributed to the larger number of anthracene groups facilitating more frequent dimerization events compared to 4-arm PEG-anthracene.

**Figure 4:**
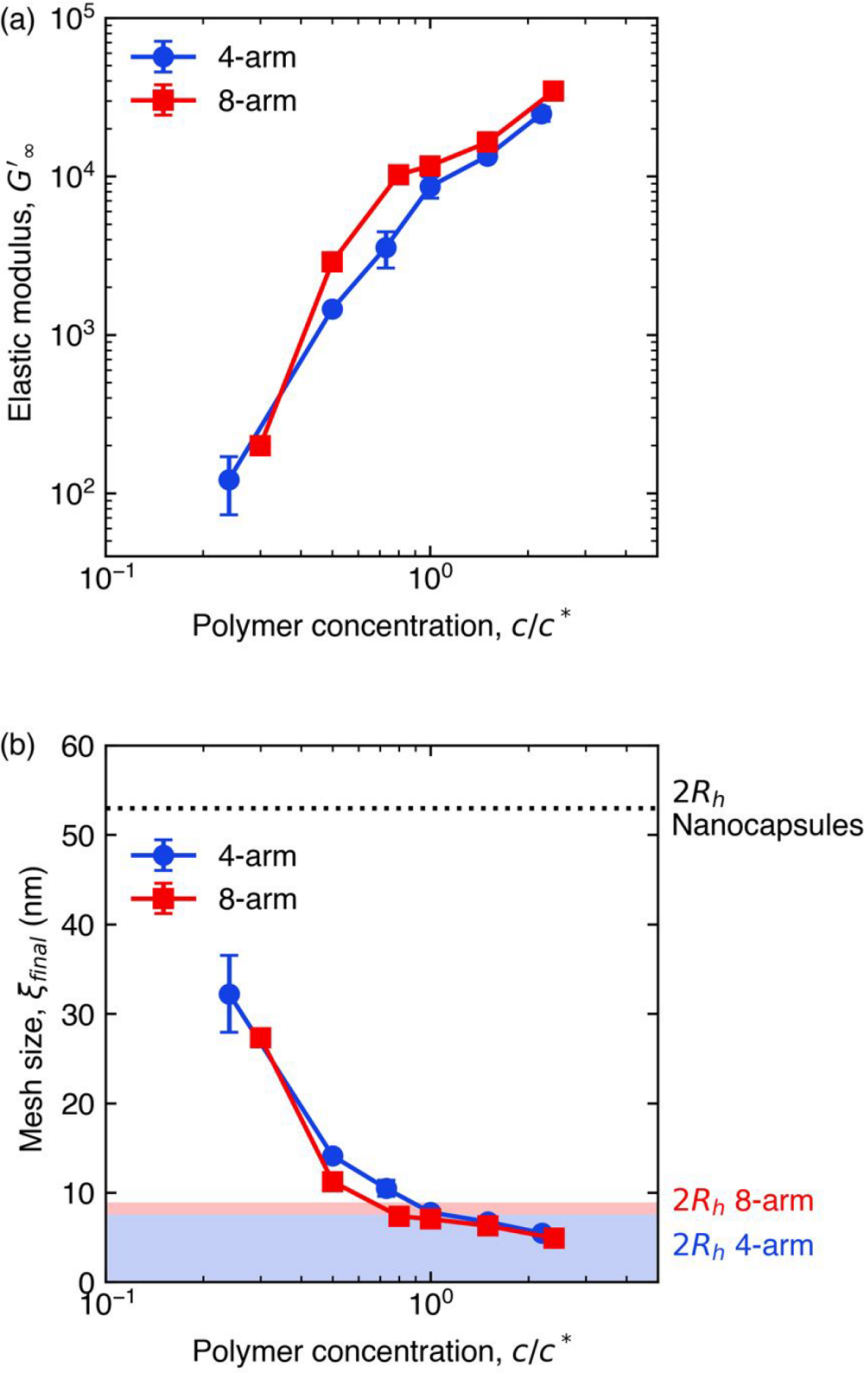
(a) 8-arm PEG-anthracene hydrogels were stiffer than 4-arm PEG-anthracene hydrogels at equivalent *c*/c* due to (b) smaller network mesh sizes determined from elastic moduli. Agreement between the network mesh size near *c*/c* = 1 and the hydrodynamic radii of multiarm PEG-anthracene (shaded regions) suggest minimal stretching of PEG-anthracene arms. Steady-state elastic moduli were determined after UV irradiation for 1 h. Error bars indicate standard error (*n* = 3), and some error bars are smaller than the symbols.

The final elastic modulus *G*_∞_^′^ indicates the extent of PEG-anthracene cross-linking and the underlying network mesh size in PEG-anthracene hydrogels. For both 4- and 8-arm PEG-anthracene, higher polymer concentrations produced hydrogels with larger *G*_∞_^′^ (Figure 4a). Generally, 8-arm PEG-anthracene hydrogels reached larger *G*_∞_^′^ than 4-arm PEG-anthracene hydrogels at comparable *c*/c*. Differences in the elastic moduli between 4- and 8-arm PEG-anthracene hydrogels are attributed to differences in arm molecular weights: 5000 g/mol for 4-arm PEG-anthracene compared to 2500 g/mol for 8-arm PEG-anthracene. The arm length determines the average distance between network junctions, such that shorter arms produce stiffer networks (larger *G*_∞_^′^). The extent of cross-linking in the hydrogel can be calculated from the number density of network junctions ν, which is proportional to the elastic modulus *G*′:^72^
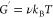
4where *k*_B_ is the Boltzmann constant and *T* is the temperature. The average distance between network junctions, or the mesh size (ξ), was calculated using the relation ξ = ν^1/3^ (Figure 4b). For steady-state, photo-cross-linked hydrogels with final elastic moduli *G*_∞_^′^, ξ decreased with increasing *c*/c* of 4- and 8-arm PEG-anthracene. At *c*/c* = 1, network mesh sizes were comparable to the hydrodynamic radii of PEG-anthracene in water, suggesting minimal stretching of PEG-anthracene arms. Above *c*/c* = 1, network mesh sizes were smaller than the hydrodynamic radii, indicating interpenetration between polymer molecules.

### 3.3 Formulation and Gelation of Polymer Nanocomposite Hydrogels

**Figure 5:**
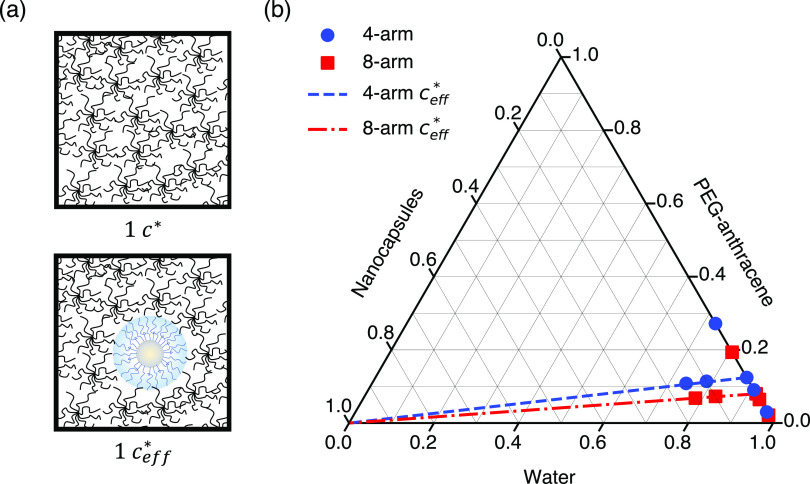
(a) Nanocapsules occupy space that is inaccessible to polymers in solution (nanocapsule and polymer molecules not drawn to scale; the nanocapsule radius is approximately six times larger than the polymer radius). (b) A ternary diagram illustrates the formulation of polymer nanocomposites comprising PEG-anthracene, silica-coated nanocapsules, and water. Each axis shows the component volumetric fraction. Symbols indicate compositions explored in experiments, and dashed lines indicate effective polymer overlap concentrations *c*_eff_^*^ due to excluded volume by nanocapsules.

The function and mechanical properties of polymer hydrogels are greatly enhanced by the incorporation of nanomaterials. In this work, polymer nanocomposite hydrogels were formulated as ternary mixtures of multiarm star polymers, silica-coated nanocapsules, and water (Figure 5). A key consideration during formulation was the spatial extent of each component in the composite material, as polymer and nanocapsule sizes dictate important length scales before, during, and after network formation. The nanocapsules used in this study had hydrodynamic radii of 26.5 ± 0.3 nm, nearly an order of magnitude larger than those of 4-arm (3.77 ± 0.04 nm) and 8-arm (4.4 ± 0.2 nm) PEG-anthracene. The different length scales between nanocapsules and polymers were important to consider in the formulation of different nanocomposites, as well as in the analysis of dynamics during photo-cross-linking to form nanocomposite hydrogels. Critically, the nanocapsules were treated as rigid spheres that occupy space inaccessible to PEG-anthracene star polymers (Figure 5a).^42,74^ To account for such excluded volume, an effective polymer overlap concentration *c*_eff_^*^ was defined according to the polymer-accessible solvent volume,
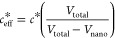
5where *V*_total_ is the sum volume of all individual components (polymer, nanocapsules, and water) and *V*_nano_ is the volume of nanocapsules in the mixture. In Figure 5b, lines of *c*_eff_^*^ for 4- and 8-arm PEG-anthracene show the reduction in polymer volume fraction required to maintain overlap as the nanocapsule volume fraction increases.

**Figure 6:**
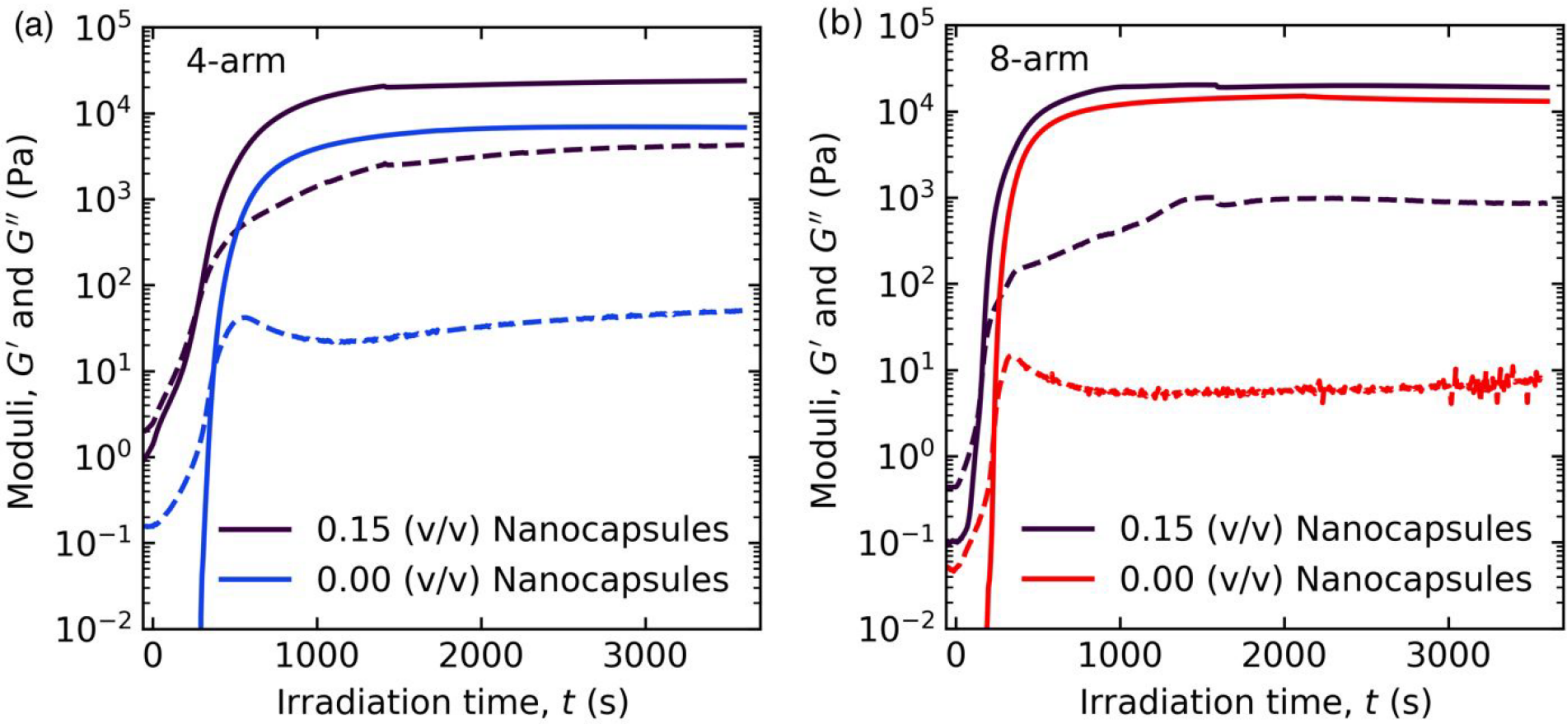
*In situ* dynamic rheology experiments reveal significant differences during the photo-cross-linking of (a) 4-arm and (b) 8-arm PEG-anthracene solutions in the presence and absence of silica nanocapsules. Nanocapsule-free solutions were measured at c*, and nanocomposites were formulated at *c*_eff_^*^. All samples were subject to irradiation with 365 nm UV light (2.7 mW/cm^2^) at 22 °C. Solid lines (—) denote the elastic modulus and dashed lines (---) denote the viscous modulus. Small-amplitude oscillatory time sweeps were conducted with strain amplitude and oscillation frequency of 10% and 10 rad/s, respectively.

The addition of nanocapsules while maintaining *c*_eff_^*^ led to faster gelation dynamics and larger final elastic moduli of PEG-anthracene hydrogels, as measured by *in situ* small-amplitude oscillatory shear rheology. Figure 6a,b illustrates significant differences during the transient network formation of PEG-anthracene hydrogels and PEG-anthracene nanocomposite hydrogels. Initially, the starting solution viscosities were higher for nanocomposite mixtures, likely due to the presence of nanocapsules. During gelation, the gel times were faster for nanocomposite mixtures relative to PEG-anthracene solutions. Elastic moduli *G*′ at the gel point were also higher for nanocomposite hydrogels, suggesting the formation of stiffer networks and potential synergistic effects from nanocapsule incorporation.^49^ Overshoots in the viscous moduli *G*″ were not observed in nanocomposite hydrogels, in contrast to PEG-anthracene hydrogels. Instead, slower rates of change in *G*″ were observed in nanocomposite hydrogels, potentially due to reduced nanocapsule mobility and caging of nanocapsules during photo-cross-linking.^75−77^ Slower rates of change in *G*″ in nanocomposite hydrogels suggest the slower growth of PEG-anthracene clusters and delayed incorporation of clusters into the polymer network. Finally, *G*_∞_^′^ values for nanocomposite hydrogels were up to 2-fold larger than for PEG-anthracene hydrogels, and *G*_∞_^″^ was over an order of magnitude larger. Drastically larger values of *G*_∞_^″^ in nanocomposites reflect liquid fractions with higher viscosities, likely due to the presence of nanocapsules. The increase in *G*_∞_^″^ may also result from nanocapsule-mediated constraints on the incorporation of PEG-anthracene clusters into the polymer network.

**Figure 7:**
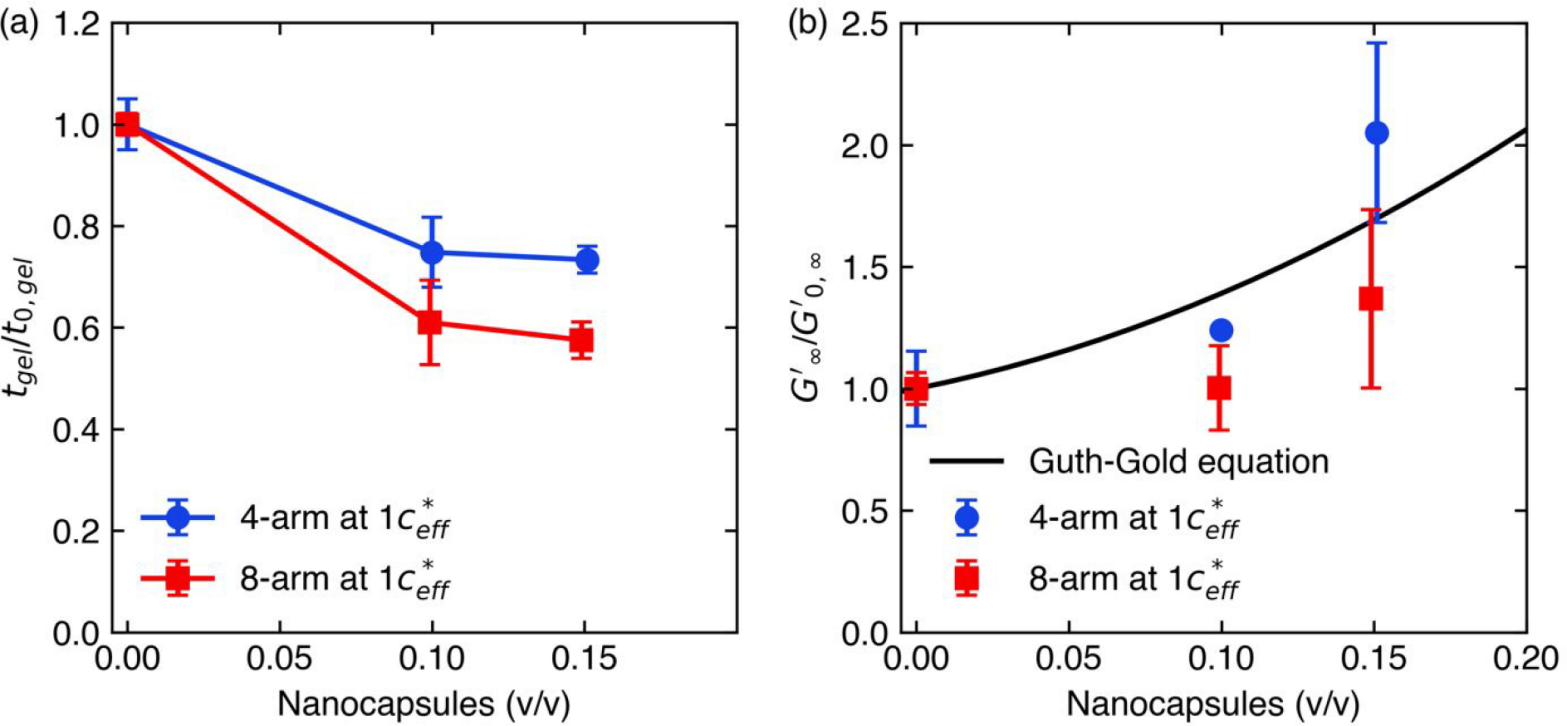
Nanocapsule addition leads to faster gelation and larger elastic moduli of PEG-anthracene nanocomposite hydrogels. (a) Gel times and (b) elastic moduli of nanocomposite hydrogels at 1 *c*_eff_^*^ are normalized to the properties of hydrogels without nanocapsules at 1 c*. The solid line in (b) represents the Guth–Gold model, which predicts an increase in elastic modulus upon addition of noninteracting nanomaterials. Error bars indicate standard error (*n* = 3), and some error bars are smaller than the symbols.

The addition of nanocapsules decreased *t*_gel_ of both 4- and 8-arm PEG-anthracene hydrogels while maintaining overlap by adjusting for *c*_eff_^*^ (Figure 7a). Adding 10% (v/v) nanocapsules led to significant decreases in *t*_gel_, and faster gelation was attributed to the reduced volume to be spanned by the polymer network upon forming a complete gel. However, the further addition of nanocapsules to 15% (v/v) only weakly decreased *t*_gel_, indicating that the reduction of the PEG-anthracene volume fraction cannot fully explain faster gelation in the presence of nanocapsules. Moreover, the decrease in *t*_gel_ upon addition of nanocapsules was nearly 2-fold greater for 8-arm PEG-anthracene nanocomposites than for 4-arm PEG-anthracene nanocomposites. This result suggests a topological dependence of polymer network formation in nanocomposites, which may arise from differences in elasticity, nanocapsule dispersion, or polymer–nanocapsule interactions in the resulting network. For example, softer networks formed by 4-arm PEG-anthracene may facilitate clustering of nanoparticles, whereas stiffer networks formed by 8-arm PEG-anthracene may facilitate more uniform dispersion of nanocapsules.^63^

Nanocapsule incorporation increased the stiffness of PEG-anthracene hydrogels (Figure 7b), despite a decrease in the true volume fraction of PEG-anthracene to maintain *c*_eff_^*^ in nanocomposite formulations. Generally, lower volume fractions of PEG-anthracene would result in fewer potential network junctions in the resulting nanocomposite hydrogel. Increased elastic moduli suggest cooperative interactions between nanocapsules and PEG-anthracene. Figure 7b shows qualitative agreement between the final elastic moduli and the Guth–Gold model (eq 2),^49^ which predicts the effect of noninteracting nanomaterials on the stiffness of polymeric materials. For 4-arm PEG-anthracene nanocomposite hydrogels, the model underpredicted the relative increase of *G*_∞_^′^ at the highest nanocapsule volume fraction. Conversely, for 8-arm PEG-anthracene nanocomposite hydrogels, the model overpredicted relative increases in *G*_∞_^′^. Discrepancies between 4- and 8-arm PEG-anthracene nanocomposites suggest potential differences in network structure; for example, nanomaterials tend to aggregate into clusters in softer networks and disperse more evenly in stiffer networks.^63^

## 4 Conclusions

*In situ* rheology was used to investigate the photo-cross-linking of polymer and polymer nanocomposite hydrogels comprising anthracene-terminated, multiarm star polymers and silica-coated nanocapsules. The time to reach the gel point upon photo-cross-linking depended on the number of arms and concentration of multiarm star polymers, with a surprising non-monotonic dependence of the gel time on polymer concentration. Non-monotonicity was attributed to different polymer concentration regimes, in which the fastest gel time was observed near the polymer overlap concentration (*c*/c* ∼ 1) due to optimal proximity of anthracene groups for photodimerization. At much lower concentrations (*c*/c* ≪ 1), slower gel times were attributed to the increased probability of intramolecular loops over intermolecular cross-links, thereby impeding the gelation process. Finally, at higher concentrations (*c*/c* > 1), slower gel times were associated with higher solution viscosities and lower frequencies of anthracene dimerization events.

The gelation dynamics and mechanical properties of photo-cross-linked polymer hydrogels were significantly altered upon the addition of silica-coated nanocapsules. Notably, faster gel times were observed upon the addition of nanocapsules, suggesting an acceleration of network connectivity due to synergistic polymer–nanocapsule interactions. Nanocapsules also increased the stiffness of polymer nanocomposite hydrogels, despite reduced polymer content to account for excluded volume by nanocapsules. Mechanical reinforcement by nanocapsule addition was more pronounced in 4-arm PEG-anthracene hydrogels than 8-arm PEG-anthracene hydrogels. The profound influence of nanocapsules on the gelation dynamics and final hydrogel properties emphasizes the importance of understanding key length scales and polymer–nanocapsule interactions in the design and formulation of nanocomposite materials.

## Supporting Information Available

^1^H NMR spectra of all functionalized polymers, schematic of custom rheo-optical setup, UV intensity calibration curve, estimation of UV penetration depth, and rheological data and analysis including oscillation amplitude sweeps, frequency sweeps before and after UV irradiation, determination of gel time, and replicate *in situ* photo-cross-linking time sweeps (PDF)
