# Supplementary material for: Gelation Dynamics during Photo-Cross-Linking of Polymer Nanocomposite Hydrogels
Source: ACS Polym Au. 2022 Dec 5;3(2):217–27. doi: 10.1021/acspolymersau.2c00051 (PMC10103194; doi:10.1021/acspolymersau.2c00051)
Supplement: Supplementary file 1 — lg2c00051_si_001.pdf [file lg2c00051_si_001.pdf]

Supplemental Information for
"Gelation dynamics during photocrosslinking of polymer nanocomposite hydrogels"

Michael C. Burroughs[1], Tracy H. Schloemer[2], Daniel N. Congreve[2], and Danielle J. Mai[1*]

[1]Department of Chemical Engineering, Stanford University, Stanford, California 94305, USA
[2]Department of Electrical Engineering, Stanford University, Stanford, California 94305, USA

[*]corresponding author; email: djmai@stanford.edu

Table of Contents

1. ^{1}H NMR confirmation of PEG-anthracene functionalization (**Figures S1-S2**)
    1.1. 4-arm, PEG-anthracene
    1.2. 8-arm, PEG-anthracene

2. Rheo-optics setup and UV intensity calibration (**Figures S3-S4**)
    2.1. Instrumentation and calibration
    2.2. Evaluation of UV penetration depth

3. Representative amplitude sweeps of post-photocured polymer and polymer nanocomposite hydrogels (**Figure S5**)

4. Frequency sweeps of polymer and polymer nanocomposite hydrogels (**Figures S6-S8**)
    4.1. 4- and 8-arm PEG-anthracene in water pre- and post-UV irradiation
    4.2. 4- and 8-arm PEG-anthracene nanocomposites pre- and post-UV irradiation
    4.3. 4- and 8-arm PEG-anthracene nanocomposites with varying nanocapsule volume fractions

5. Determination of gel time during photocrosslinking (**Figure S9**)

6. Representative *in situ* small-amplitude oscillatory shear time sweeps (**Figure S10**)

1. ^{1}H NMR confirmation of PEG-anthracene functionalization

1.1. <u>4-arm, 20K PEG-anthracene</u>: 0.441 g (85% yield); ^{1}H NMR (500 MHz, $CDCl_3$) δ 8.47 (s, 1H), 8.10 (d, *J* = 8.7 Hz, 2H), 8.02 – 7.97 (m, 2H), 7.49 (dd, *J* = 13.1, 6.9 Hz, 4H), 6.83 (t, 1H), 3.64 (s, 448H).

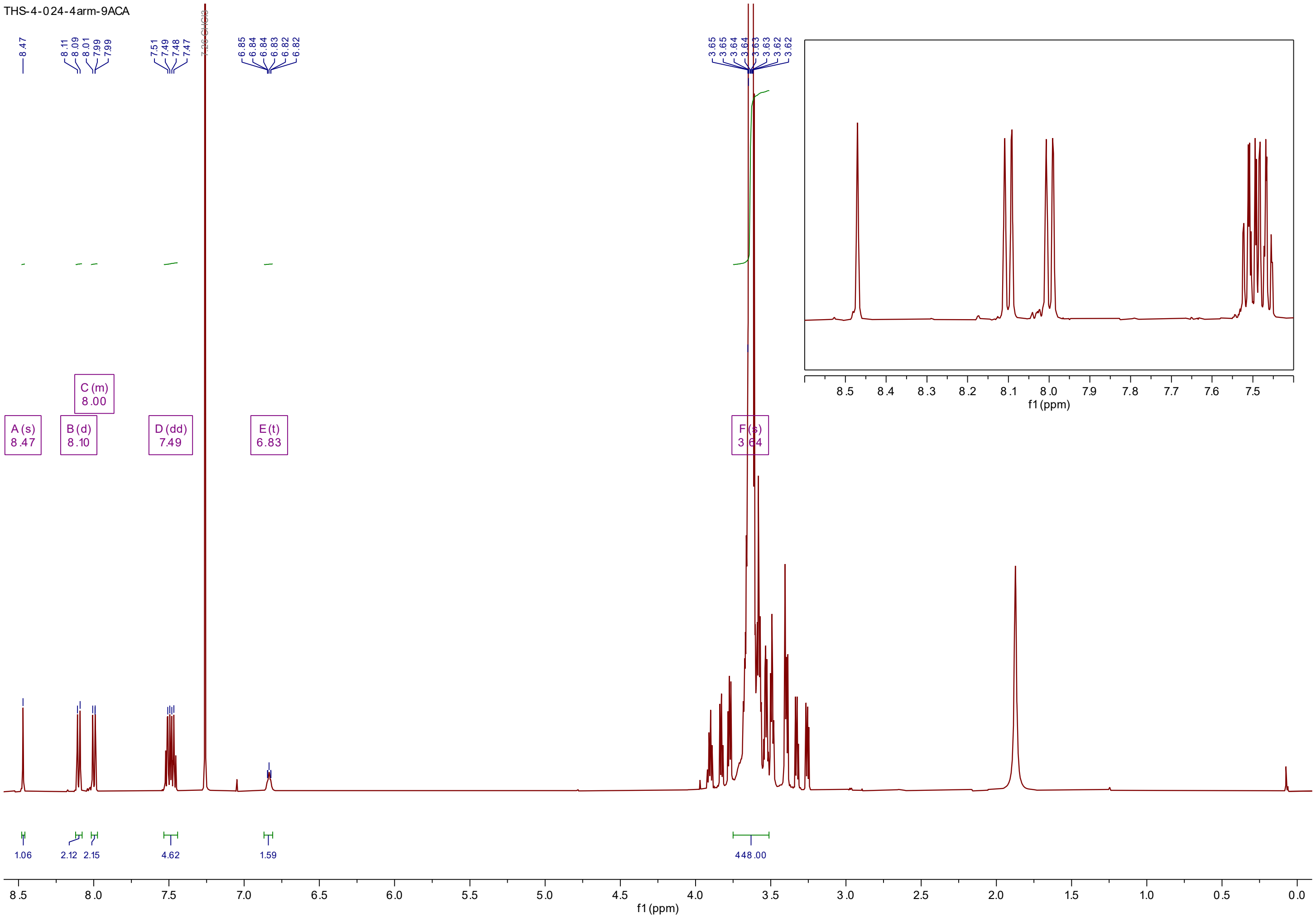

Figure S1. ^{1}H NMR spectra of 4-arm PEG-anthracene in $CDCl_3$.

1.2. 8-arm, 20K PEG-anthracene: 0.514 g (95% yield), ^{1}H NMR (500 MHz, $CDCl_3$) δ 8.45 (s, 1H), 8.08 (d, J = 8.7 Hz, 2H), 7.98 (d, J = 7.9 Hz, 2H), 7.47 (dddd, J = 19.2, 7.9, 6.5, 1.3 Hz, 4H), 6.82 (t, J = 5.6 Hz, 1H), 3.62 (s, 448H).

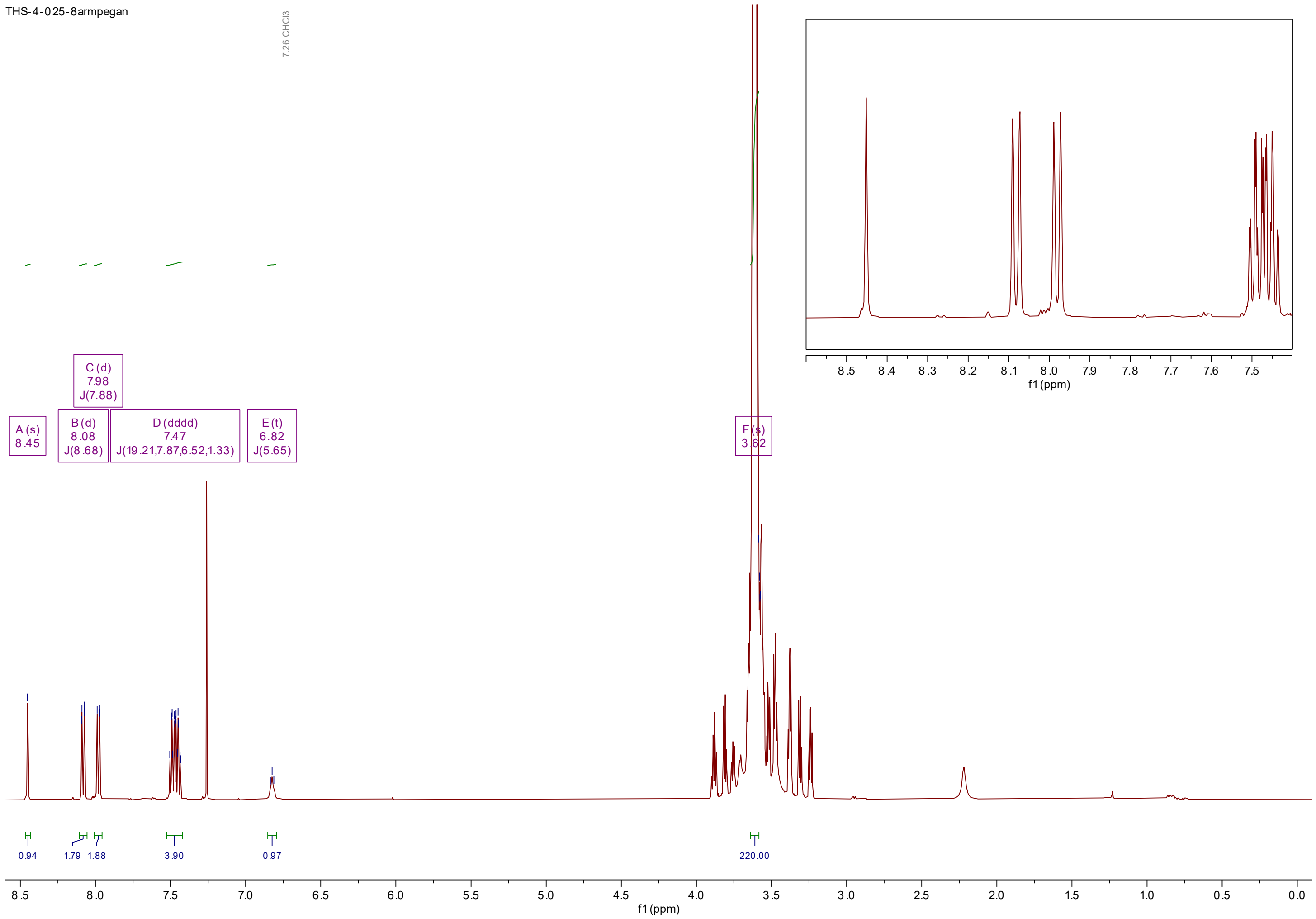

Figure S2. H NMR spectra of 8-arm PEG-anthracene in $CDCl_3$.

2. Rheo-optical setup and calibration

2.1. Instrumentation and calibration

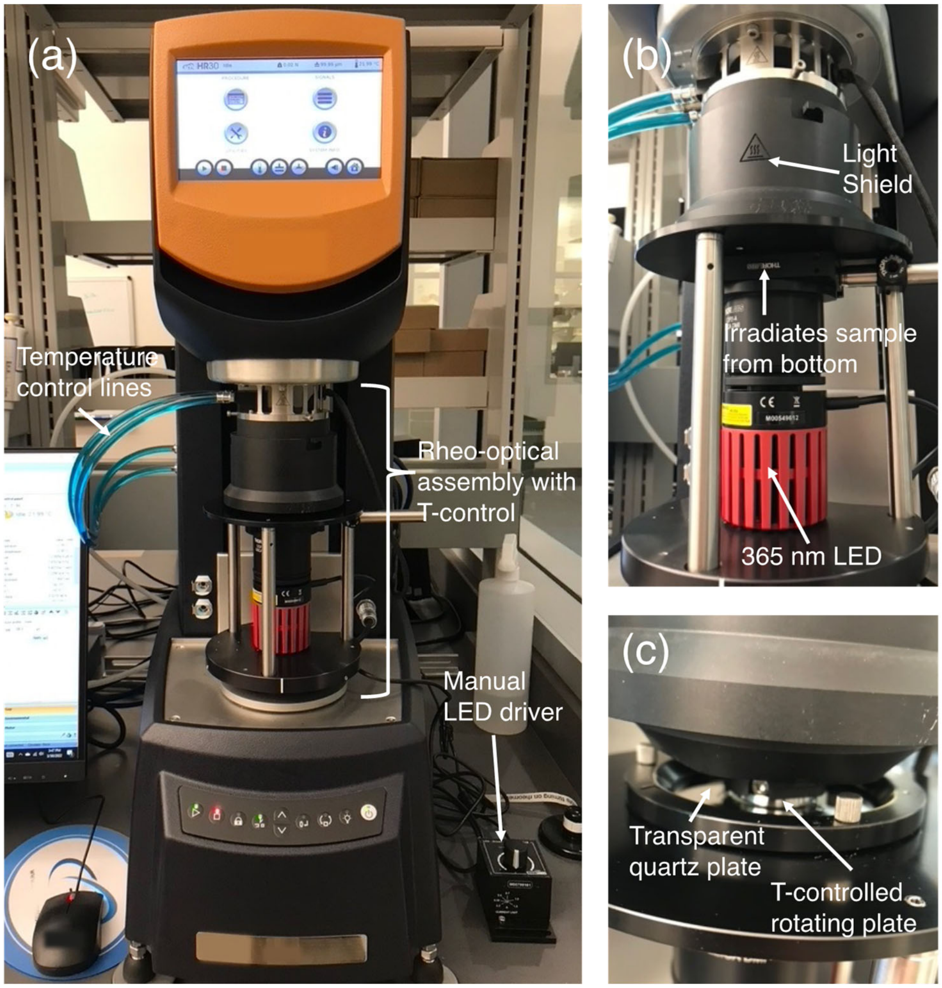

Figure S3. Photographs of the rheo-optical setup with component labels for (a) the total assembly, (b) the optical train, and (c) the transparent quartz bottom plate and temperature-controlled upper plate that confine the sample during rheological measurements.

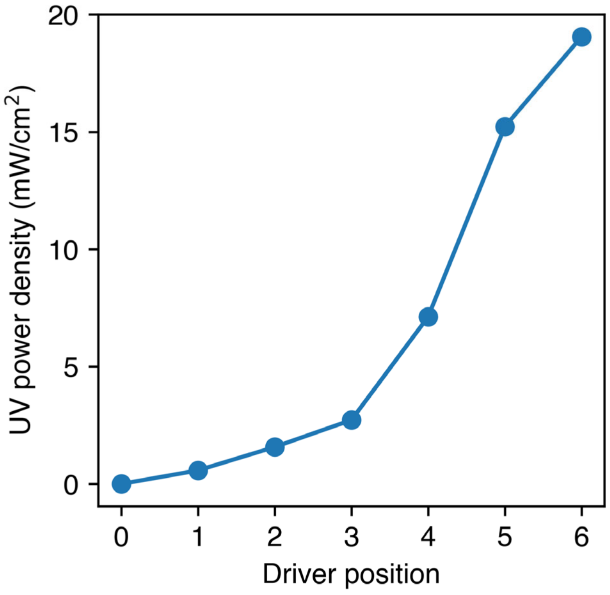

Figure S4. Calibration of UV power density to knob position on manual LED driver. Power density was measured using a ThorLabs PM100D power meter with a S120VC sensor (200-1100 nm). All photocrosslinking experiments were performed at a driver position of '3'.

2.2. Evaluation of UV penetration depth

UV light has notoriously low penetration depths through solution. In preliminary experiments, we observed increases in the crossover time with increased sample thickness, which indicates a thickness dependence of PEG-anthracene crosslinking using our custom setup. To circumvent this challenge, we conducted all measurements reported in the manuscript at a constant gap of 100 µm. This gap thickness is the lowest recommended thickness for our system.

To assess the UV light penetration across our sample, we neglect the time dependencies of molar absorptivity, scattering, and photoactive species concentration by estimating spatial intensity variations according to the governing differential equation:

$$\frac{\partial I(z)}{\partial z} = -2.3 I(z) \varepsilon_{\mathrm{anthracene}} [c_{\mathrm{anthracene}}]$$

where I is the irradiated light intensity, z is the sample thickness (0.01 cm), $\varepsilon_{anthracene}$ is the molar absorptivity of anthracene at 365 nm (assumed to be 3125 cm^{-1} M^{-1}), and $[c_{anthracene}]$ is the concentration of anthracene functional groups (estimated as 0.03 M). Assuming the boundary condition of $I(z = 0) = I_0$, we can solve the differential equation:

$$\frac{I}{I_0} = \exp(-2.3 \varepsilon_{\mathrm{anthracene}} [c_{\mathrm{anthracene}}] z).$$

Using this equation, we estimate light to attenuate by nearly 90% across the initial sample thickness. We note that this is a worst-case scenario given the fact that as the reaction proceeds, the molar absorptivity decreases to nearly zero, thereby reducing the light attenuation across the sample to be negligible for fully crosslinked materials.

3. Oscillatory amplitude sweeps of photocrosslinked polymer and polymer nanocomposite hydrogels

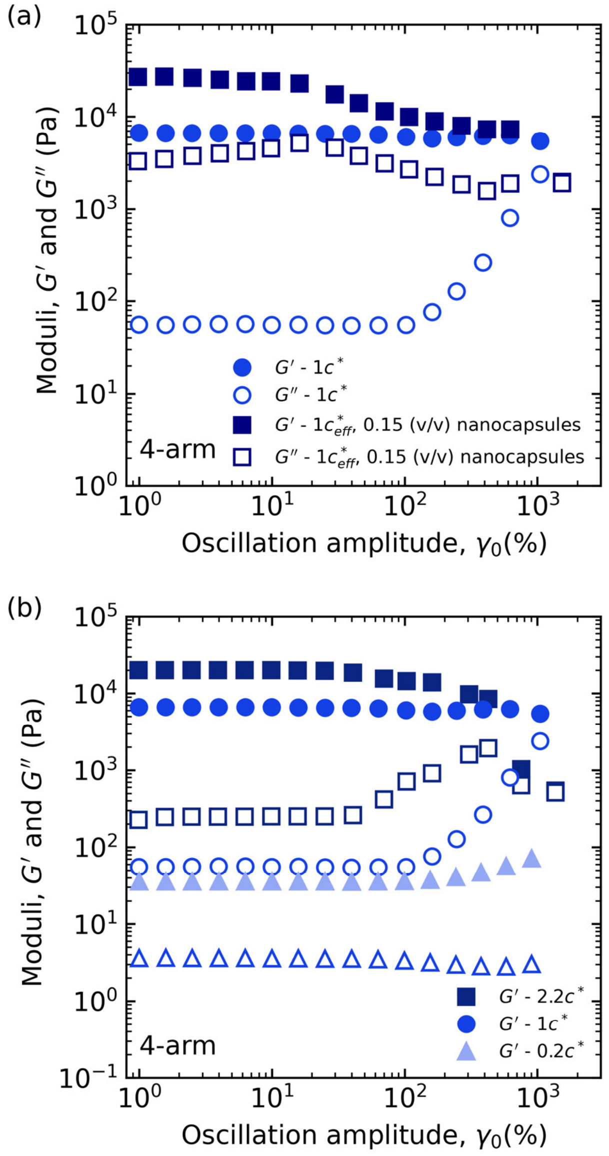

Figure S5. Oscillatory amplitude sweeps (a) with and without nanocapsules and (b) for varying polymer solution concentrations to confirm rheological measurements remain in the linear viscoelastic regime. Oscillation frequency for all measurements was held fixed at 10 rad/s.

4. Frequency sweeps of polymer and polymer nanocomposite hydrogels

2.1. 4- and 8-arm PEG-anthracene in water pre- and post-UV irradiation

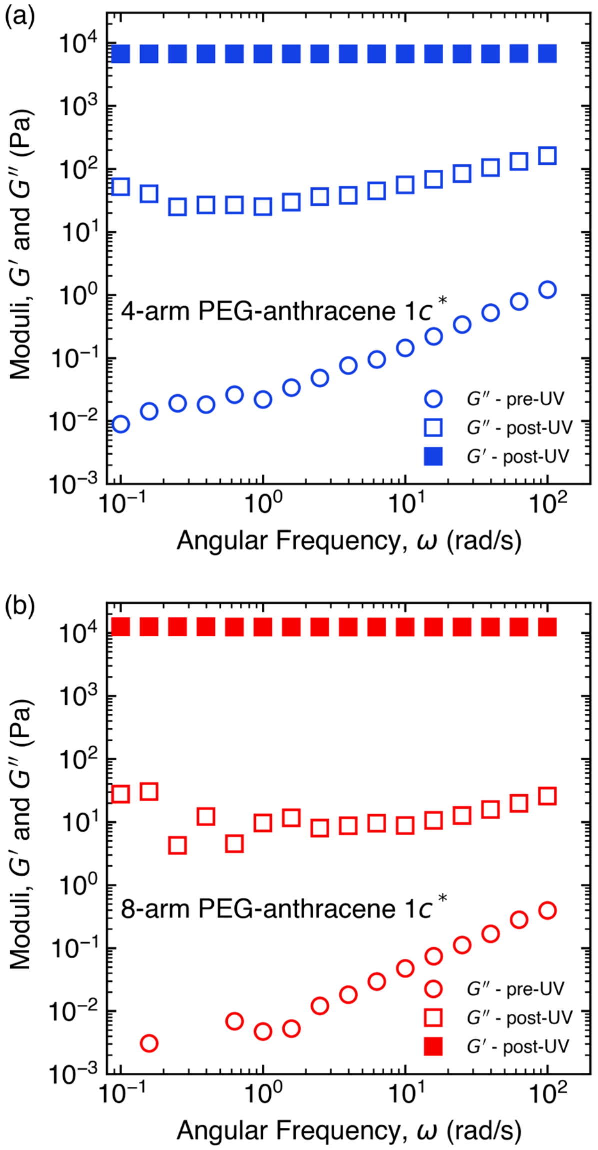

Figure S6. Frequency sweeps of (a) 4-arm and (b) 8-arm PEG-anthracene solutions before and after UV irradiation. Measurements were performed at 10% strain amplitude and 22 °C.

2.2. 4- and 8-arm PEG-anthracene nanocomposites pre- and post-UV irradiation

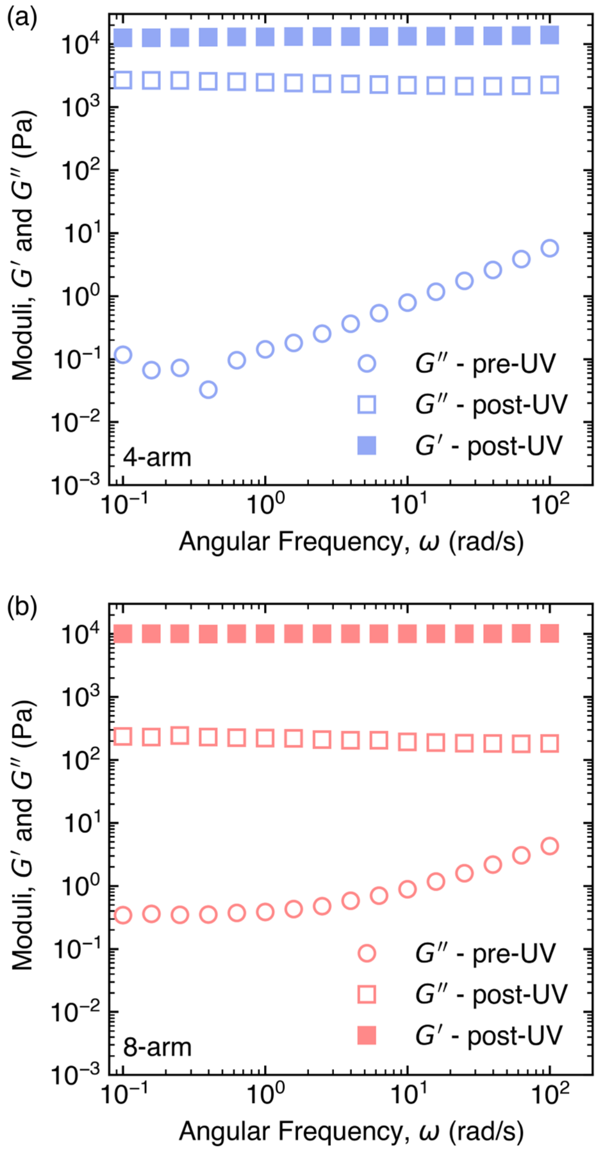

Figure S7. Frequency sweeps of (a) 4-arm and (b) 8-arm PEG-anthracene nanocomposites (1 c_{eff}^{*}) with 15% (v) nanocapsules before and after UV irradiation. Measurements were performed at 10% strain amplitude and 22 °C.

2.3. 4- and 8-arm PEG-anthracene nanocomposites with varying nanocapsule volume fractions

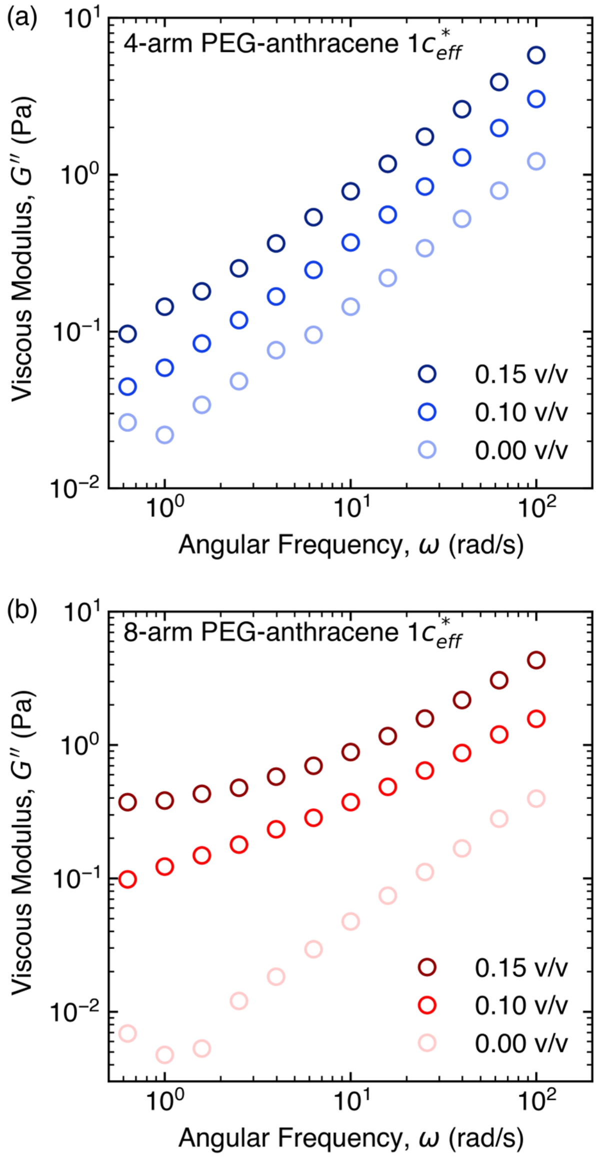

Figure S8. Frequency sweeps of (a) 4-arm and (b) 8-arm PEG-anthracene nanocomposites with varying nanocapsule loadings before UV irradiation. Measurements were performed at 10% strain amplitude and 22 °C.

5. Determination of the gel time during photocrosslinking

The gel time was determined from the G'', G' crossover time measured during PEG-anthracene photocrosslinking. The time at which G'' and G' crossover is equivalent to the time at which $\tan(\delta) = 1$, since $G''/G' = \tan(\delta)$. Time-dependent $\tan(\delta)$ data in the vicinity of $\tan(\delta) = 1$ were fit to a single exponential decay function of the form $y(x) = a * \exp(-b * x) + c$. To ensure that the experimental data could be approximated by a single exponential decay function, time was rescaled by subtracting the early time data outside of the span used for curve fitting. Optimized values of $a, b,$ and c were determined using the curve_fit function from the scipy.optimize library of Python. The gel time was then determined by solving $1 = a * \exp(-b * x) + c$ for x and adding back the time subtracted for curve fitting purposes.

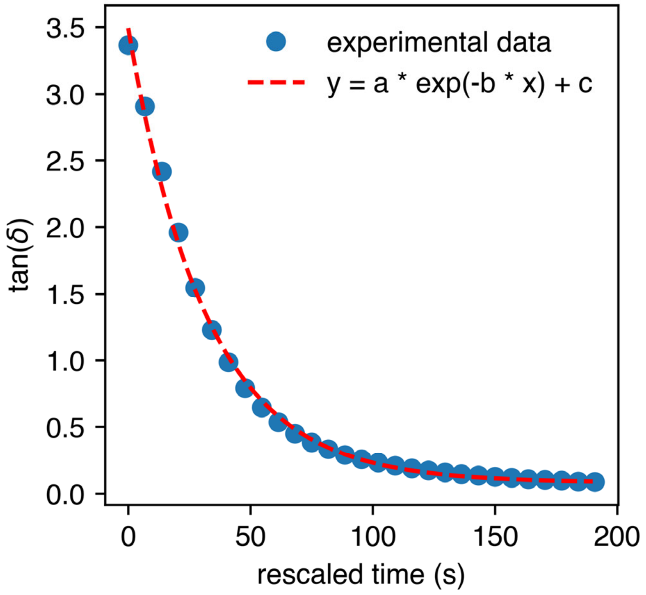

Figure S9. Example curve fitting process for determining the gel time from small-amplitude oscillatory shear experimental data.

6. Representative replicate *in situ* small-amplitude oscillatory shear time sweeps

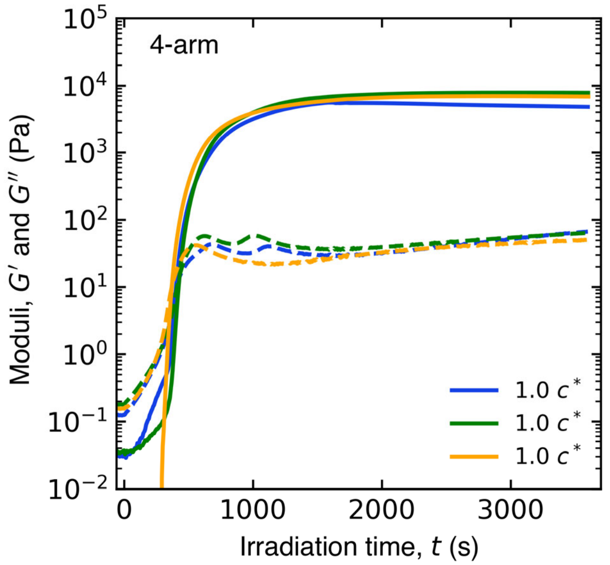

Figure S10. Replicate *in situ* small-amplitude oscillatory time sweeps show variability in the number of overshoots in G'' during photocrosslinking.
